# Computational fluid dynamics study of intra-arterial chemotherapy for oral cancer

**DOI:** 10.1186/s12938-017-0348-5

**Published:** 2017-05-15

**Authors:** Hiroaki Kitajima, Marie Oshima, Toshinori Iwai, Yoshihito Ohhara, Yasuharu Yajima, Kenji Mitsudo, Iwai Tohnai

**Affiliations:** 1 Department of Oral and Maxillofacial Surgery, Yokohama City University Graduate School of Medicine, 3-9 Fukuura, Kanazawa-ku, Yokohama, 236-0004 Japan; 2 Interfaculty Initiative in Information Studies, The University of Tokyo, 7-3-1 Hongo, Bunkyo-ku, Tokyo, 113-0033 Japan; 3 Institute of Industrial Science, The University of Tokyo, 4-6-1 Komaba, Meguro-ku, Tokyo, 153-8505 Japan

**Keywords:** Oral cancer, Intra-arterial chemotherapy, Computational fluid dynamics, Simulation, Blood flow, External carotid artery and its branches

## Abstract

**Background:**

Intra-arterial chemotherapy (IAC) for oral cancer can deliver a higher concentration of anticancer agent into a tumor-feeding artery than intravenous systemic chemotherapy. However, distribution of anticancer agent into several branches of the external carotid artery (ECA) in IAC has not demonstrated sufficient treatment efficacy. To improve the effectiveness of IAC, the flow distribution of anticancer agent into the branches of the ECA in several IAC methods was investigated using computational fluid dynamics (CFD).

**Methods:**

Patient-specific three-dimensional vessel models were created from CT images of 2 patients with tongue cancer. Catheter models were combined with the vessel models. Thirty-two models were generated with varying vertical and horizontal positions of the catheter tip. With the use of a zero-dimensional resistance model of the peripheral vessel network, conventional IAC and superselective IAC were simulated in 30 and 2 models, respectively. The flow distribution of anticancer agent into the branches of the ECA was investigated in 32 models. Additionally, the blood streamline was traced from the inlet of the common carotid artery toward each outlet to examine the flow of anticancer agent in all models, and the wall shear stress of the vessel was calculated for some models.

**Results:**

The CFD simulations could be conducted within a reasonable computational time. In several models, the anticancer agent flowed into the target artery only when the catheter tip was located below the bifurcation of the ECA and each target artery. Furthermore, the anticancer agent tended to flow into the target artery when the catheter tip was shifted toward the target artery. In all ECA branches that had flow of anticancer agent, the blood streamlines to the target arteries contacted the catheter tip. Anticancer agent flowed into only the target artery in patients’ models for superselective IAC. However, high wall shear stress was observed at the target artery in one patient’s model.

**Conclusions:**

This CFD study showed that location of the catheter tip was important in controlling the anticancer agent in conventional IAC. The distribution rate of anticancer agent into the tumor-feeding artery tended to increase when the catheter tip was placed below and toward the target artery. Although superselective IAC can reliably supply anticancer agent to the target artery, high wall shear stress at the target artery can occur, depending on vessel geometry of the patient, which may cause serious complications during the treatment.

## Background

In the treatment of oral cancer, surgery is considered to be the most effective curative modality. However, radical surgery for advanced oral cancer often causes severe oral dysfunction—such as disorders of speech, mastication, and swallowing—and results in lower quality of life. To preserve organ function, a multidisciplinary approach that combines radiotherapy and chemotherapy has become a viable alternative to radical surgery in advanced cases [1, 2].

**Fig. 1:**
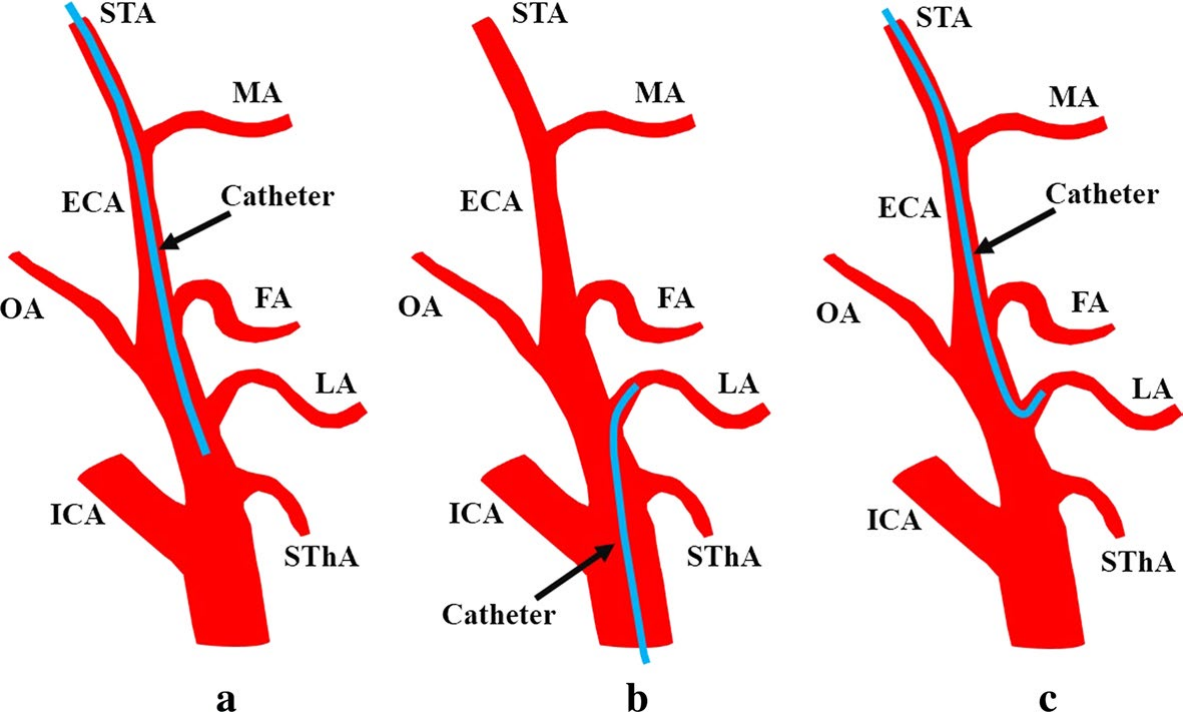
Catheterization method for intra-arterial chemotherapy. Three types of intra-arterial infusion are shown for a case of cancer of the tongue that is fed by the lingual artery. **a** Conventional intra-arterial chemotherapy via the superficial temporal artery (STA). **b** Superselective intra-arterial chemotherapy via the femoral artery. **c** Superselective intra-arterial chemotherapy via the STA. *ECA* external carotid artery, *FA* facial artery, *ICA* internal carotid artery, *MA* maxillary artery, *OA* occipital artery, *SThA* superior thyroid artery

Intra-arterial chemotherapy (IAC) has been used to treat head and neck cancer since the 1950s [1]. In this method, a linear catheter is placed near a target tumor-feeding artery originating from the external carotid artery (ECA; Fig. 1a). Intratumoral concentrations of anticancer agents therefore fluctuate, and the efficacy of early IAC was unproven [1, 3]. Recently, progress in vascular radiological techniques has led to the development of superselective IAC (SSIAC), which has the advantage of delivering a higher concentration of anticancer agent to the tumor bed than conventional IAC, and has been applied to head and neck cancer, including oral cancer [1–3]. SSIAC may be delivered via the superficial temporal artery (STA) or femoral artery (Fig. 1b, c) [1, 3], and a catheter is inserted into a tumor-feeding artery originating from the ECA, such as the lingual artery (LA), facial artery (FA), or maxillary artery (MA). In SSAIC via the femoral artery (Seldinger’s method), the catheter is inserted into a branch of the ECA in an antegrade fashion by interventional radiologists (Fig. 1b) [1, 3–5]. Because the catheter is inserted through the common carotid artery (CCA) when an anticancer agent is injected, severe complications such as cerebral infarction and sudden death have been reported, albeit rarely [3–5]. In contrast, SSAIC via the STA does not always require interventional radiologists, and a hook-shaped catheter (Medikit Co., Ltd, Tokyo, Japan) can be inserted into the target artery in a retrograde fashion by oral and maxillofacial surgeons or otolaryngologists (Fig. 1c) [3, 6, 7]. However, when catheter insertion into the target tumor-feeding artery is difficult, the catheter is placed in the ECA and conventional IAC is performed, resulting in unpredictable flow of anticancer agents [6].

In IAC via the STA, the concentration of anticancer agents introduced to branches of the ECA is unclear. In our previous study of IAC for patients with tongue cancer [7], concentrations of an anticancer agent (carboplatin) were measured in resected tumor tissues after IAC and SSIAC. The mean platinum concentration in tumor tissue after IAC was about 40% of the amount delivered after SSIAC (4.3 ± 3.8 μg/g wet vs 10.5 ± 1.2 μg/g wet, respectively). The standard deviation of platinum concentration after IAC was larger and in some cases, the anticancer agent rarely flowed via the LA (tumor-feeding artery). This study showed that the concentration of anticancer agent delivered to the tumor-feeding artery was more unpredictable after IAC than after SSIAC.

Although it is useful to simulate the distribution of anticancer agent in the ECA for each patient, there are few reports on the application of computational fluid dynamics (CFD) to IAC analysis in head and neck cancer, including oral cancer [8, 9]. To apply CFD as a tool to assist treatment, simulations must be conducted accurately within a reasonable computational time. For realistic blood flow simulation, the pressure condition is particularly important because it determines the flow distribution among the arteries. In our previous study [9], we developed a model for the outflow boundary condition as a zero-dimensional (0D) resistance model of the peripheral vasculature, which consists of only resistance to be incorporated into the commercial three-dimensional (3D) flow simulation software. The new 0D model could provide physiological pressure at each outlet and allow accurate blood flow simulation in the ECA and its branches. In the present study, the boundary condition given in [9] was modified to simulate the flow of blood and anticancer agent in the ECA and its branches. The purpose of this CFD study was to numerically investigate the flow distribution of anticancer agent into the branches of the ECA in several IAC methods with the aim of improving the effectiveness of IAC.

## Methods

### Patient-specific geometric model

#### Computed tomography angiography

Patients with oral cancer underwent computed tomography (CT) angiography before IAC. We used a 64-slice CT scanner (Aquilion 64; Toshiba Medical Systems, Tokyo, Japan), and non-ionic contrast medium (100 mL) was injected at a rate of 4.0 mL/s through an antecubital vein with an automatic power injector. A bolus-tracking technique was used to individually select the scan delay of the arterial phase. Repetitive low-dose scans were performed with a delay of 8 s at a level inferior to the carotid bifurcation. To measure the bolus arrival time, the region of interest was chosen to lie in the CCA. The scanning procedure started automatically when an enhancement level of 90 Hounsfield units was reached. For the arterial phase scan, the scanning volume included the inferior margin of the thyroid cartilage/bottom of C6 and the superior margin of the orbit. The scanner settings were 120 kV, 250 mA, slice collimation 64 × 0.5 mm, table speed 20.5 mm/rotation (pitch 0.641), and rotation time 0.75 s. The resolution matrix was 512 × 512 pixels with a slice thickness of 1 mm.

We investigated 3D-CT angiography of the ECA and its branches in many patients with oral cancer and selected 2 patients with tongue cancer with origins in the LA, FA, and occipital artery (OA), which are orthogonal to or aligned with one another in distal view of the ECA.

#### Patient-specific model

**Fig. 2:**
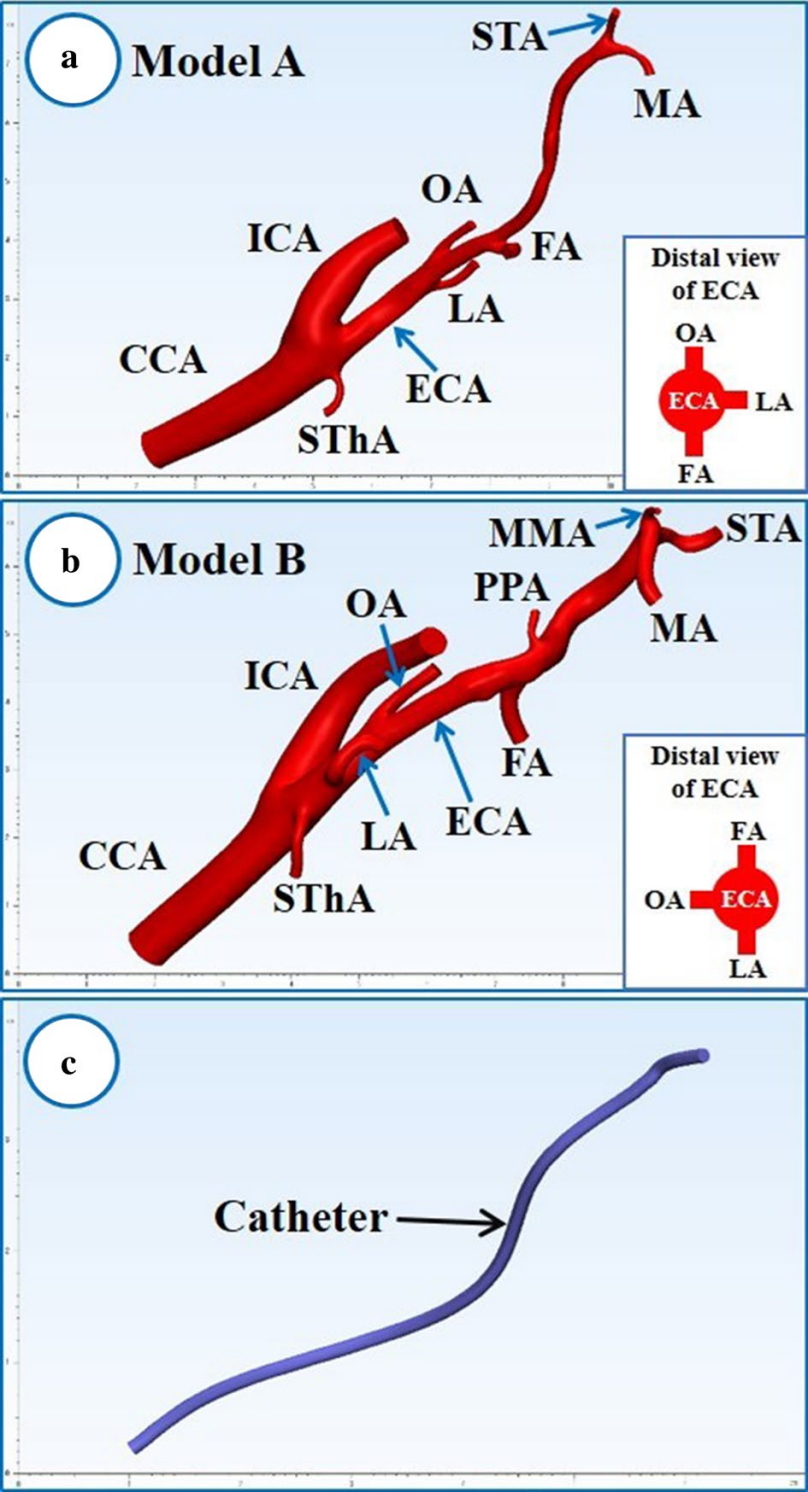
Three-dimensional vessel models and catheter. **a** Three-dimensional model of the right carotid artery and its branches in case A. **b** Three-dimensional model of the left carotid artery and its branches in case B. **c** Three-dimensional catheter model (diameter: 1.3 mm). *ECA* external carotid artery, *FA* facial artery, *ICA* internal carotid artery, *LA* lingual artery, *MA* maxillary artery, *MMA* middle meningeal artery, *OA* occipital artery, *SThA* superior thyroid artery

**Fig. 3:**
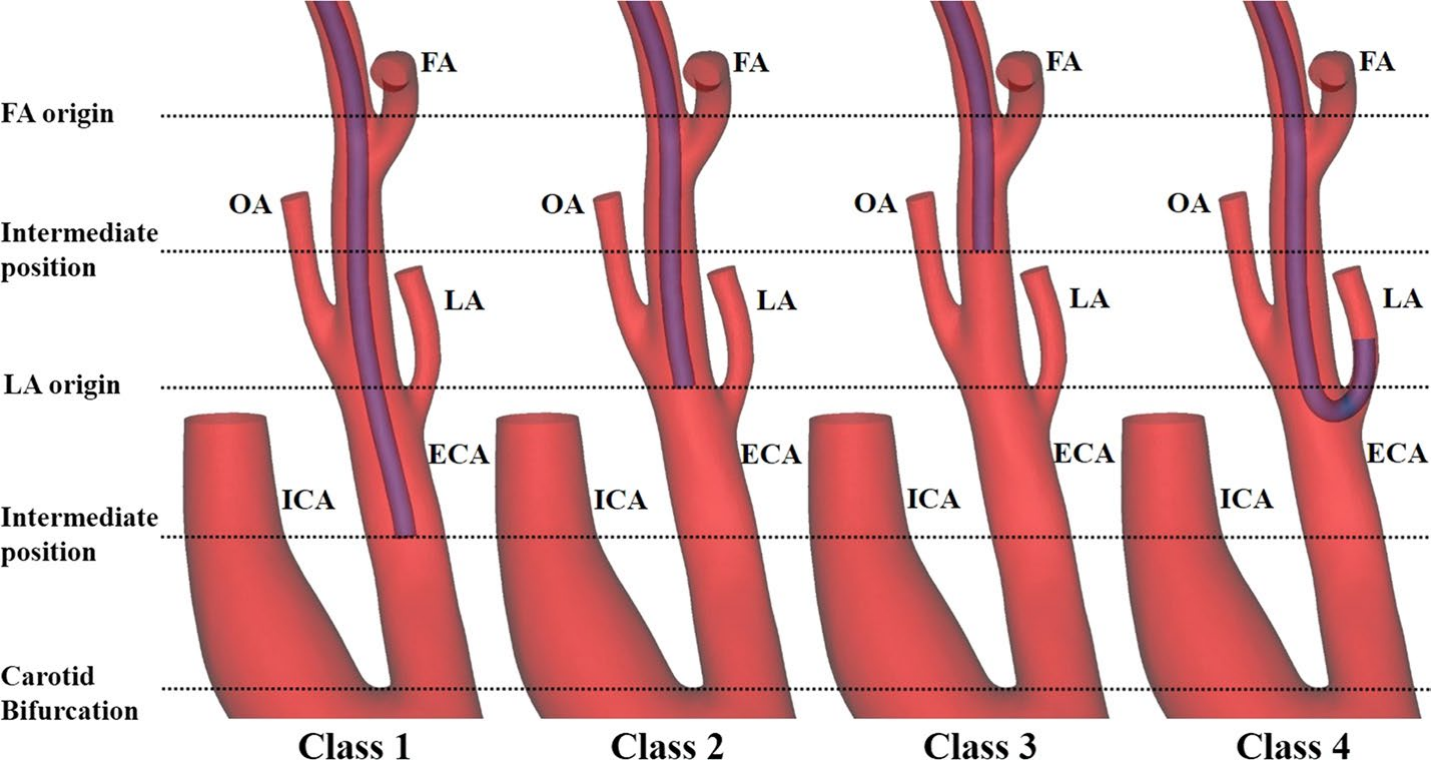
Classification of vertical position of catheter tip. *ECA* external carotid artery, *FA* facial artery, *ICA* internal carotid artery, *LA* lingual artery, *MA* maxillary artery, *MMA* middle meningeal artery, *OA* occipital artery

**Fig. 4:**
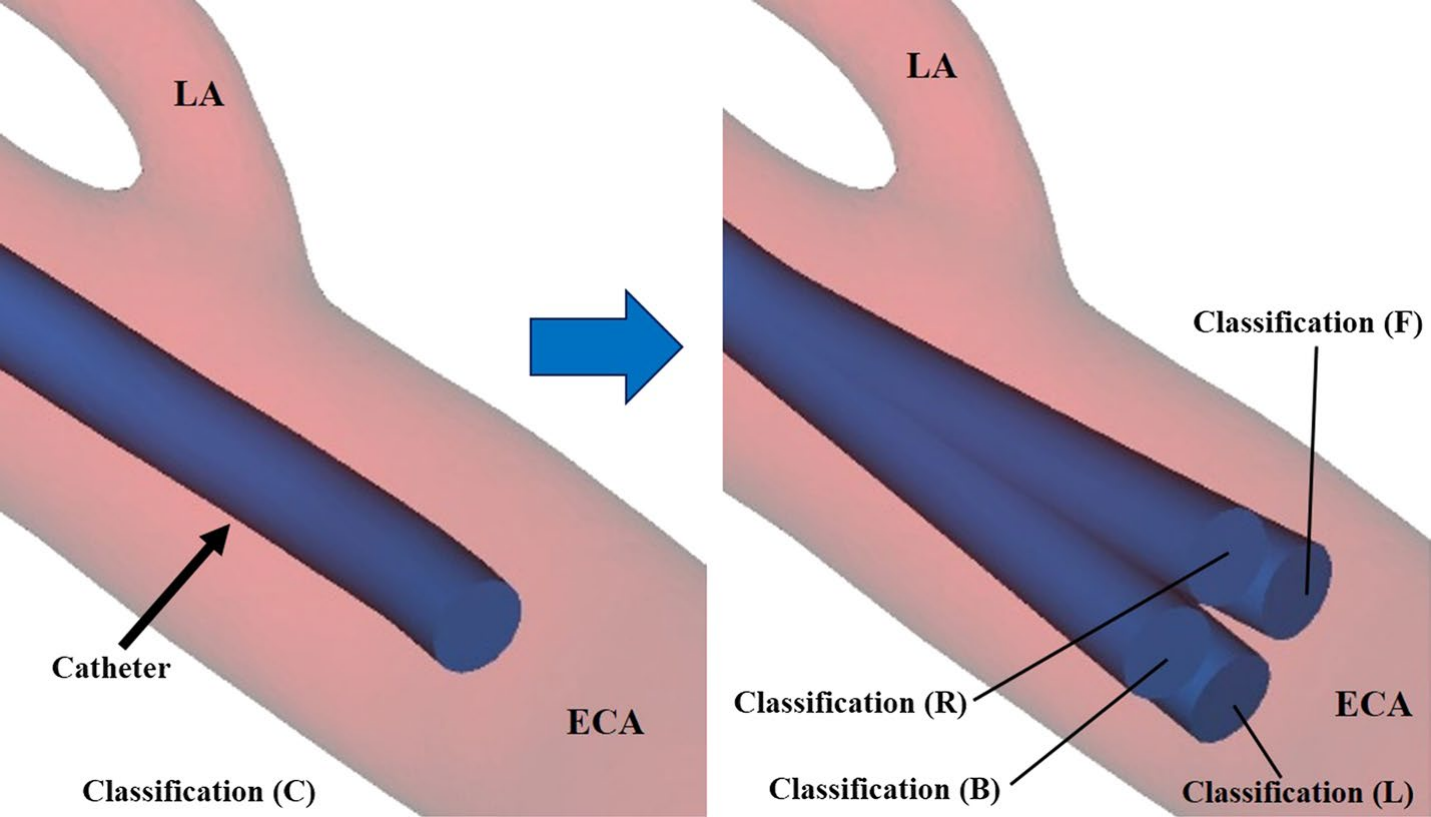
Classification of* horizontal* position of catheter tip. Target arteries are the lingual, facial, and occipital arteries. The catheter tip rotates centering on a point 10 mm away from the tip, and the amount of shift from the centerline of the catheter is a quarter of the diameter of the external carotid artery (ECA). Classification of* horizontal* position of catheter tip is determined in distal view of the ECA. *LA* lingual artery

Digital Imaging and Communications in Medicine (DICOM) data from CT angiography of the 2 patients with tongue cancer were input into Mimics software (Materialise, Leuven, Belgium). The carotid artery and its branches were segmented, and 2 patient-specific 3D geometrical models with origins in the LA, FA, and OA were created (Fig. 2a, b). The models included the CCA, internal carotid artery (ICA), and ECA branches such as the superior thyroid artery (SThA), LA, OA, FA, posterior auricular artery (PAA), middle meningeal artery (MMA), MA, and STA. Because of CT resolution, model A did not include the PAA or MMA. According to our previous study [9], each artery was cut off at a length five times its diameter to minimize the influence of boundary conditions on the flow distributions. In addition, a 1.3-mm–diameter catheter, which is in clinical use, was designed in Standard Triangulated Language (STL) format (Fig. 2c). The catheter model was combined with the vessel model for IAC via the STA using Mimics software. Thirty-two models were generated with variations in the vertical and horizontal positions of the catheter tip (Figs. 3, 4; Tables 1, 2).

**Table 1:** Classification of vertical position of catheter tip

Class	Position of catheter tip
1	Intermediate position between the carotid bifurcation and LA origin (bifurcation between the ECA and LA)
2	LA origin (bifurcation between the ECA and LA)
3	Intermediate position between the LA origin and FA origin (bifurcation between the ECA and FA)
4	Catheter insertion into the LA (superselective intra-arterial catheterization)

*ECA* external carotid artery, *FA* facial artery, *LA* lingual artery

**Table 2:** Classification of horizontal position of catheter tip

Class	Position of catheter tip
C	Center of the ECA
F	Forward shift of catheter tip toward the target artery from center of the ECA
B	Backward shift of catheter tip toward the target artery from center of the ECA
L	Left shift of catheter tip toward the target artery from center of the ECA
R	Right shift of catheter tip toward the target artery from center of the ECA

Target arteries are the lingual, facial, and occipital arteries. Catheter tip rotates centering on a point 10 mm away from the tip, and the amount of shift from the centerline of the catheter is a quarter of the diameter of the external carotid artery (ECA). Classification of horizontal position of catheter tip is determined in distal view of the ECA

#### Mesh generation

**Fig. 5:**
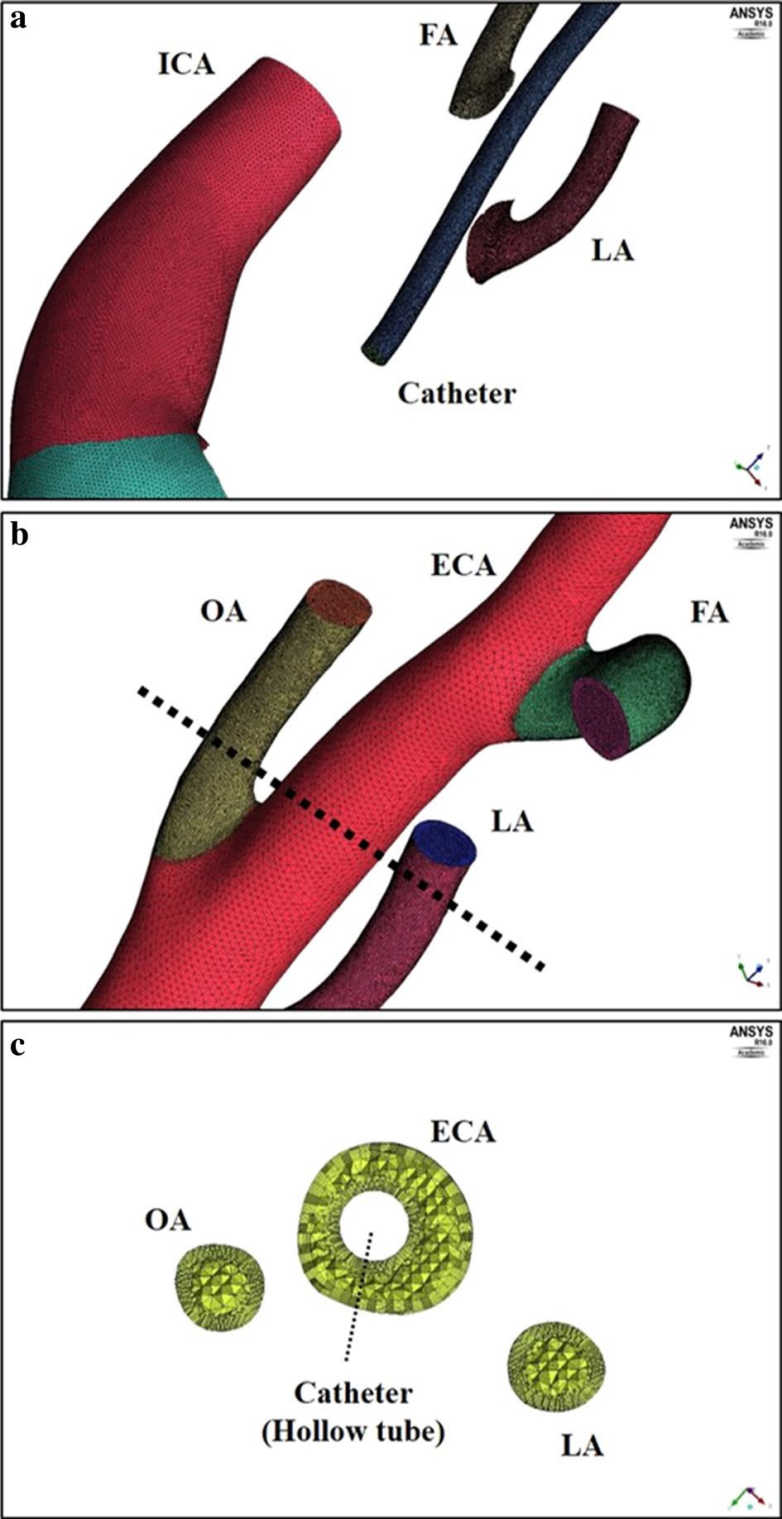
Surface and volume mesh for model A1-C. **a** The surface mesh of the vessel and catheter for model A1-C. The wall of the ECA is hidden. **b** The surface mesh of the vessel. **c** The volume mesh of the vessel. Cross-sectional view of the *dot*-*line* in **b**. *ECA* external carotid artery, *FA* facial artery, *ICA* internal carotid artery, *LA* lingual artery, *MA* maxillary artery, *MMA* middle meningeal artery, *OA* occipital artery

Computational grids were created from the surface of the geometrical model. The volume meshes were generated using ICEM-CFD software (Ansys Inc., Canonsburg, PA) for each analysis model. Figure 5 shows the surface and volume meshes of vessel and catheter for model A1-C. The mesh consists of tetrahedral cells in the center region and prismatic cells in the region near the arterial wall and the catheter wall (Fig. 5a, b, respectively). Mesh sensitivity tests were performed for models A1-C and B1-C. For mesh sensitivity tests, mass flow rate of the anticancer agent (kg/s) at each outlet was compared among different mesh densities ranging from 1,320,913 to 8,072,925 cells for model A1-C and from 5,459,786 to 18,971,746 cells for model B1-C. Mesh density was adjusted until the mass flow rate of anticancer agent at each outlet was less than 1.32 × 10^‐7^ (kg/s) (1.0% of the distribution rate of the anticancer agent). As a result, the adopted numbers of cells for models A1-C and B1-C were 5,713,318 and 16,885,069, respectively. The same cell sizes for each part in models A1-C and B1-C were applied for 15 other models in cases A and B, respectively, and the average number of cells was 5,424,908 in case A and 16,467,416 in case B. The catheter was assumed to be a hollow tube without any inside meshes (Fig. 5c) and the boundary condition of anticancer agents was prescribed as the inflow boundary condition, because anticancer agents are injected at a constant velocity using a syringe driver and recirculation cannot occur in the catheter during intra-arterial infusion.

### Numerical methods

Anticancer agents were assumed to be transported along with the blood. Species transport analysis was conducted using FLUENT software (Ansys Inc.) to simulate the intra-arterial infusion together with blood flow analysis. The flow fields of the mixture were solved using the mass and the momentum conservation equations of incompressible single-phase flow, while the flow of the anticancer agent was solved as a function of the concentration (mass fraction) of the advection–diffusion Eq. (1) as follows:

1 $$\frac{\partial }{\partial t}\left( {\mathop \rho \nolimits_{m} \mathop Y\nolimits_{i} } \right) + \nabla \cdot \left(\mathop \rho \nolimits_{m} \overrightarrow {v} \mathop Y\nolimits_{i} \right) = - \nabla \cdot \overrightarrow {{\mathop F\nolimits_{i} }}$$

where *Y* is the concentration (mass fraction) of the species, subscript *i* indicates the species number, and species numbers 0 and 1 represent the anticancer agent and blood, respectively. The concentration of the anticancer agent (*Y*_*0*_) was calculated from Eq. (1). As the sum of the mass fractions of the species is always 1, the concentration of blood (*Y*_*1*_) is calculated as the difference between 1 and *Y*_*0*_. *ρ*_*m*_ is the density (kg/m^3^) of the mixture and is described below. *F*_*i*_ is the diffusion flux of the species caused by the concentration gradient. Dilute approximation (Fick’s law) was used to model the mass diffusion caused by the concentration gradient, and the diffusion flux is expressed in Eq. (2) on the basis of the following approximation:

2 $$\overrightarrow {{\mathop F\nolimits_{i} }} = - \mathop \rho \nolimits_{m} \mathop D\nolimits_{i,m} \nabla \mathop Y\nolimits_{i}$$

where *D*_*i,m*_ is a diffusion coefficient (m^2^/s) of species *i* in the mixture. In this study, the anticancer agent was assumed to be a continuum of water. Although there is currently no report on the measurement values of the diffusion coefficient of water or of the anticancer agent to blood, diffusion coefficients between liquids are reported in the order of approximately 0.25 × 10^−9^ to 4.56 × 10^−9^ m^2^/s at temperatures of 8–30 °C [10]. In this study, the self-diffusion coefficient of water at 25 °C (2.299 × 10^−9^ m^2^/s) [11] was used as the diffusion coefficient of water to blood. The density (*ρ*_*m*_) and viscosity (*μ*_*m*_) of the mixture were defined as functions (3) and (4) of the mass fraction of species *i*:

3 $$\rho_{m} = \frac{1}{{\mathop {\sum\nolimits_{i} {\frac{{Y_{i} }}{{\rho_{i} }}} }\nolimits }}$$

4 $$\mu_{m} = \sum\limits_{i} {Y_{i} \mu_{i} }$$

Regarding the physical properties of each species, the anticancer agent has a density (*ρ*_*0*_) and viscosity (*μ*_*0*_) of 998.2 kg/m^3^ and 0.0010 Pa s, respectively, whereas the values for blood (*ρ*_*1*_ and *μ*_*1*_) are 1050.0 kg/m^3^ and 0.0046 Pa s, respectively. In this study, the viscosity of blood (*μ*_*1*_) was considered constant within the 3D analysis domain, whereas *μ*_*1*_ was defined by hematocrit and the diameters of the blood vessels in the 0D resistance model used for the outlet boundary conditions (see Sect. "Modeling blood viscosity").

In practice, anticancer agents are injected through the catheter for 1 h at a constant velocity using a syringe driver. Because this study focused on the average flow distribution of the anticancer agent to the arteries, steady-state fluid dynamics simulations using the finite volume FLUENT software were performed without the pulsation of blood flow. A double-precision solver was utilized for all models. A SIMPLE (Semi-Implicit Method for Pressure-Linkage Equations) scheme was used to couple velocity and pressure, and the discretization was accurate to the second order. The 0D resistance model, which we previously developed for the outflow boundary condition [9], was used as input data for user-defined functions in the code. The convergence of the simulations was verified by monitoring the mass flow rate (kg/s) of the anticancer agent at each outlet, and was achieved when the monitored value showed oscillations of the order of 10^−9^ or less.

Fluid dynamic simulations were performed on a PC running the Microsoft Windows 7 Professional operating system (Microsoft Corp., Redmond, WA). The CPU was a dual-core Intel Xeon W5590 (clock frequency: 3.33 GHz), and there were 64 GB of RAM per core. Serial computing with one core for model A and parallel computing with 4 cores for model B were performed because model B has a larger number of cells than model A.

### Mathematical modeling of the outflow boundary conditions

#### Geometrical modeling of the peripheral vessel network

Because the flow of the anticancer agent is affected by blood flow in the ECA, accurate blood flow is required in the 3D model. To achieve this, we developed a boundary condition for the 0D resistance model to simulate blood flow in the ECA and its branches [9], which assumed a peripheral vessel network. In preliminary analyses, the flow of the anticancer agent was simulated in models A1-C and B1-C without the outflow boundary condition. In the analyses, zero-pressure was given at each outlet. The simulation result was compared with that when using the 0D resistance model. The mass flow rates, distribution rates of the anticancer agent at each outlet (ECA branch) with two outflow boundary conditions, and resistance values in the 0D model are shown in Tables 3 and 4. Because there were different results between the two outflow boundary conditions and it has been demonstrated that the 0D resistance model can provide accurate outlet pressure (see Ohhara [9]), we decided to use the 0D resistance model for this study.

**Table 3:** Mass flow rate (×10^−6^ kg/s), distribution rate of the anticancer agent, and resistance value (mmHg/kg/s) in model A1-C

Artery	A1-C		
	Mass flow rate with 0D model	Mass flow rate without 0D model	Resistance value in 0D model
ICA	0 (0%)	0 (0%)	17,790
SThA	0 (0%)	0 (0%)	386,370
LA	0 (0%)	0.2 (1.5%)	276,843
OA	0 (0%)	0.1 (0.8%)	320,828
FA	9.2 (69.7%)	12.6 (95.5%)	139,474
MA	4.0 (30.3%)	0.3 (2.3%)	313,216

*ICA* internal carotid artery, *LA* lingual artery, *MA* maxillary artery, *MMA* middle meningeal artery, *OA* occipital artery, *SThA* superior thyroid artery

**Table 4:** Mass flow rate (×10^−6^ kg/s), distribution rate of the anticancer agent, and resistance value (mmHg/kg/s) in model B1-C

Artery	B1-C		
	Mass flow rate with 0D model	Mass flow rate without 0D model	Resistance value in 0D model
ICA	0 (0%)	0 (0%)	19,432
SThA	0 (0%)	0 (0%)	293,588
LA	7.5 (56.8%)	11.1 (84.1%)	121,408
OA	0 (0%)	0.1 (0.8%)	155,639
FA	1.6 (12.1%)	1.4 (10.6%)	98,513
PAA	0.3 (2.3%)	0.2 (1.5%)	508,917
MA	0.3 (2.3%)	0.4 (3.0%)	105,510
MMA	3.5 (26.5%)	0 (0%)	1,742,672

*ICA* internal carotid artery, *FA* facial artery, *LA* lingual artery, *MA* maxillary artery, *MMA* middle meningeal artery, *OA* occipital artery, *PAA* posterior auricular artery, *SThA* superior thyroid artery

**Fig. 6:**
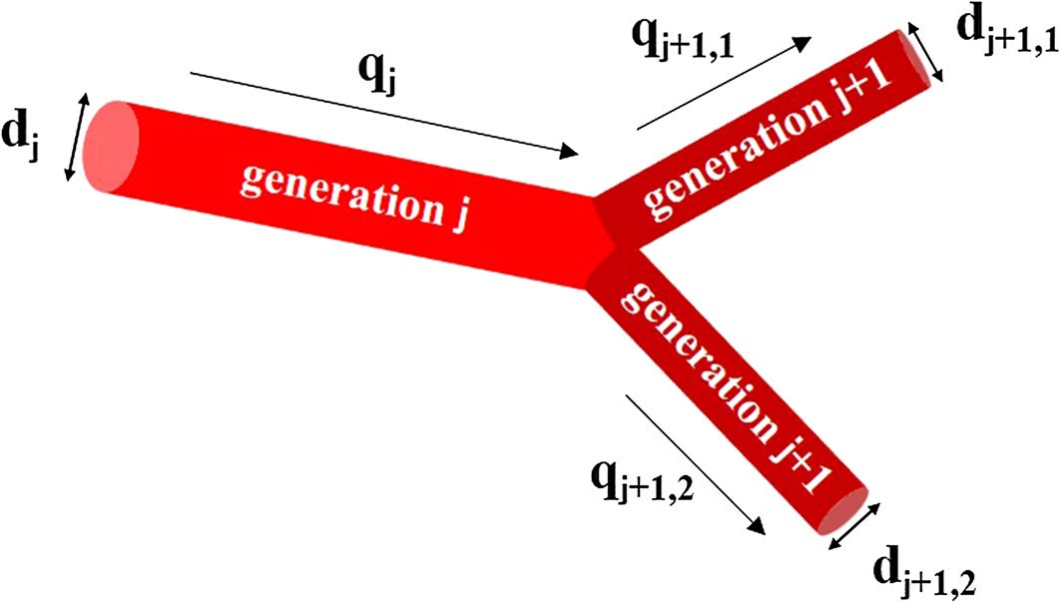
Vessel diameter and quantity of flow in peripheral network. As the parent vessel bifurcates into 2 daughter vessels, the number of generations increases. The parent blood vessel of generation *j* has diameter *d*_*j*_, and the flow rate *q*_*j*_ is equally divided into 2 branches

Because the resolution of CT or magnetic resonance imaging is not sufficient for accurate geometric modeling of a peripheral blood vessel network with small arteries, arterioles, and capillaries, the peripheral network was constructed by self-similarity geometry. The parent blood vessel of generation *j* has diameter *d*_*j*_, and the flow rate *q*_*j*_ is divided into 2 branches in 2 daughter generations (*j* + *1, 1*) and (*j* + *1, 2*), as shown in Fig. 6. The peripheral network is categorized into 6 groups according to vessel diameter, as described in [9]: (1) ICA, (2) large arteries, (3) main artery branches, (4) terminal artery branches, (5) arterioles, and (6) capillaries (Table 5).

**Table 5:** Diameter, length, and ratio of vessel length to vessel radius (*λ*) of each vessel in present peripheral network

Blood vessel	Mean diameter (mm)	Min. diameter (mm)	Length (mm)	*λ*
Internal carotid artery	5.4834	4	117.3925	50.0154
Large arteries	6.5	2	200	61.5385
Main artery branches	2.4	1.2	100	83.3333
Terminal artery branches	1.2	0.1	10	16.6667
Arterioles	0.1	0.015	2	40
Capillaries	0.008	0.004	1	250

#### 0D resistance model

Similar to our previous study [9], we defined the ratio of vessel length to vessel radius *λ* = *l/r*, where *l* is the length and *r* is the radius of a blood vessel. We can express the relationship between the pressure drop and flow rate in the time domain as follows:

5 $$\frac{{P_{n} \left( L \right)}}{{Q_{n} \left( L \right)}} = R_{n} \left( L \right)$$

where *P*, *Q*, and *R* denote the pressure, quantity of flow, and resistance, respectively. The suffix *n* denotes the *n*th generation of bifurcation.

The resistance model consisted of two steps. The first was calculation of the resistance *Rj* (0) at the inlet of the artery of the *j*th generation:

6 $$R_{j} \left( 0 \right) = \frac{{8\mu_{m} \lambda }}{{\left( {\pi r_{j}^{3} } \right)}} + R_{j} \left( L \right)$$

where *μ*_*m*_ is the viscosity of the mixture of the anticancer agent and blood as described later, *λ* is the ratio of the vessel length to its radius, and *Rj (L)* is the resistance at the outlet of the *j*th generation, in which *L* is the distance from the inlet to the outlet of the *j*th generation.

The second step was computation of the resistance *Rj (L)* at the outlet of the artery of the *j*th generation:

7 $$R_{j} (L) = \frac{1}{2}R_{j + 1,1} \left( 0 \right)$$

The peripheral network was categorized into 6 groups based on vessel diameter and length, as previously described, and the average value of *λ* for each group was determined. If the radius of the artery at the *j*th generation indicated that the vessel was in the *k*th group, *λ*_*j*_ was given by the value of *λ* for the *k*th group as shown in Table 5. Moreover, the terminal resistance was given by:

8 $$\mathop R\nolimits_{n} \mathop {(L)}\nolimits_{terminal} = \frac{{\mathop P\nolimits_{terminal} }}{{\mathop Q\nolimits_{n} \mathop {(L)}\nolimits_{terminal} }}$$

**Fig. 7:**
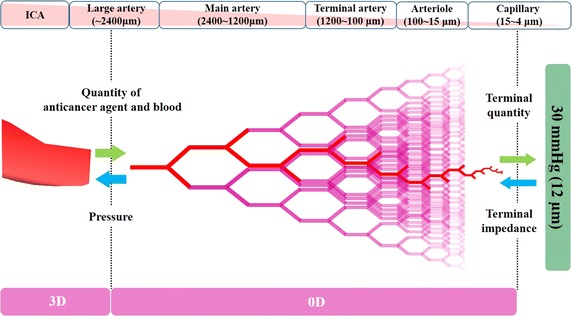
Data passing between three-dimensional outlet and zero-dimensional resistance model. Outlet pressure is calculated according to the mass flow rate at each outlet in the 3D outlet. The mass fraction of anticancer agent and blood are both constant. The viscosity of blood is defined as a function of vessel diameter and hematocrit. *0D* zero-dimensional, *3D* three-dimensional, *ICA* internal carotid artery

Equation (8) allowed us to update the terminal resistance at every step to match the physiological conditions of *P*_*terminal*_. The terminal minimum radius of the model was defined as 12 μm, and the pressure was set at 30 mmHg (Fig. 7) based on the findings of our study [9].

In the previous study [9], we performed blood flow simulation using the 0D resistance model and considered the viscosity of blood as the viscosity of the model. However, in the present study, the mixture of blood and anticancer agent was simulated and therefore the viscosity in our resistance model depended on the composition of the blood and anticancer agent as defined by formula (4). Moreover, assuming that the mass fraction of the anticancer agent *(Y*_*0*_) and the blood (*Y*_*1*_ = *1−Y*_*0*_) were constant in the 0D resistance model, *Y*_*0*_ and *Y*_*1*_ were treated with only one value. As preliminary analyses, the anticancer agent was simulated in A1-C and B1-C using the two conditions below:

1. The averaged nodal value of *Y*_*0*_ at each outlet was used for *Y*_*0*_ in the 0D resistance model.
2. *Y*_*0*_ was not considered in the 0D resistance model. In this condition, the fluid in the 0D model was treated as blood, and the *μ*_*app*_ detailed in Sect. "Modeling blood viscosity" was used for viscosity.

When comparing the simulation results under the two conditions, there were few differences at each outlet into which the anticancer agent flowed. Therefore, we decided to use the average *Y*_*0*_ nodal value.

### Modeling blood viscosity

In large and medium arteries (3D region), blood behaves as an incompressible Newtonian fluid. In contrast, blood exhibits non-Newtonian behavior in small branches and capillaries [9, 12]. To take into account non-Newtonian effects, the apparent viscosity is given as a function of both the diameter and the hematocrit [13–16]. First, the hematocrit is given by

9 $$Hct = \left\{ {\begin{array}{*{20}c} {0.45} \\ {0.45(0.196\,\,\log d - 0.117)} \\ \end{array} \,} \right.\,\,\begin{array}{*{20}c} \quad{\text{if}} \\ \quad{\text{if}} \\ \end{array} \,\,\,\begin{array}{*{20}c} {d > 300\,\mu m} \\ {d \le 300\,\mu m} \\ \end{array}$$

where *d* is the vessel diameter.

When the vessel diameter is smaller than 10 μm, the apparent viscosity decreases in accordance with the Fåhraeus–Lindqvist effect. By the inverse Fåhraeus–Lindqvist effect, the apparent viscosity increases when the vessel diameter is larger than a certain value. Equation (9) incorporates both effects.

**Fig. 8:**
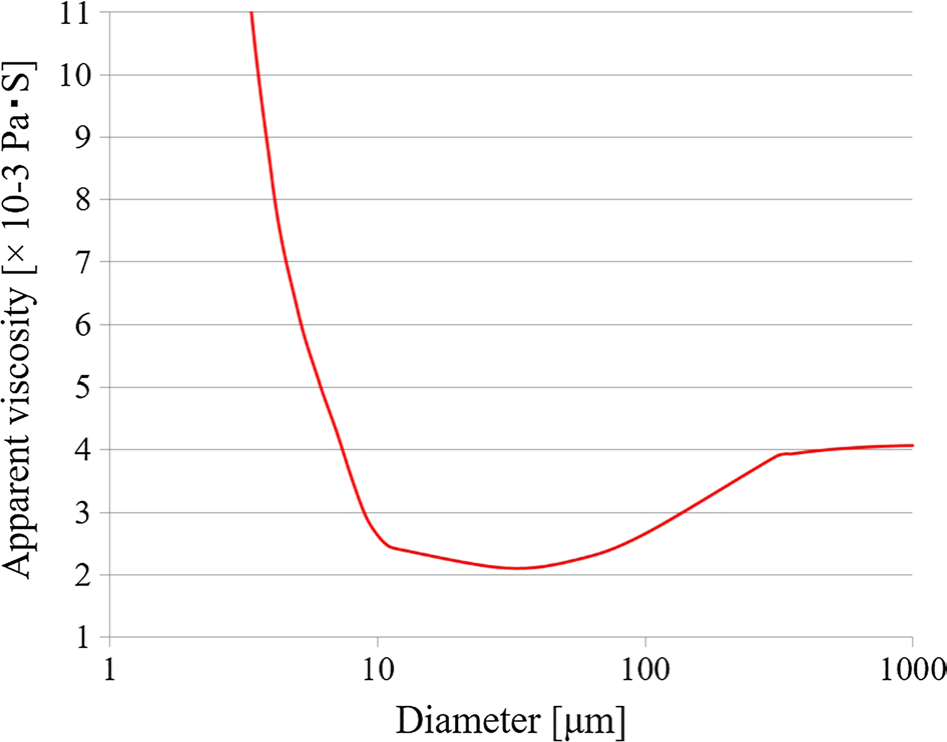
Change in apparent viscosity of blood with diameter of the vessel

The red line in Fig. 8 shows the behavior of apparent viscosity in this study, which matches that in previous studies [16, 17]. We used Eq. (10), which was proposed by Pries et al. [17, 18], to obtain the following relationship between the viscosity *μ*_*app*_ and vessel diameter *d*.

10 $$\mu_{app} = \left[ {1 + \left( { \mu_{app} - 1} \right)\frac{{\left( {1 - Hct} \right)^{cd} - 1}}{{\left( {1 - 0.45} \right)^{cd} - 1}}\left( {\frac{d}{d - 1.1}} \right)^{2} } \right]\left( {\frac{d}{d - 1.1}} \right)^{2}$$

where

11 $$Cd = \left( {0.8 + \exp \left[ { - 0.075d} \right]} \right)\left( { - 1 + \frac{1}{{1 + 10^{ - 11} d^{12} }}} \right) + \frac{1}{{1 + 10^{ - 11} d^{12} }}$$

and

12 $$\mu_{0.45} = 6\exp \left[ { - 0.085d} \right] + \mu_{Newtonian} - 2.44\exp \left[ { - 0.06d^{0.645} } \right]$$

When *μ* (the viscosity of the hematocrit) is 0.45, *μ*_*Newtonian*_ is considered to be 4.06 × 10^−3^ (Pa s). In the 0D resistance model,* μ*_*app*_ was substituted for μ_1_ in formula (4).

### Numerical conditions

Each analysis model has 2 velocity inlets (the inlet of the CCA and the catheter tip) and 6 or 8 pressure outlets. Considering the inflow boundary conditions, the blood flow velocity at the CCA was measured by ultrasonography in each patient before catheterization. The mean velocities were obtained by averaging 4 consecutive cardiac cycles and were 0.38 m/s in case A and 0.43 m/s in case B. The velocity distributions were calculated using the Poiseuille flow. A uniform distribution velocity was calculated from the flow rate given at an administration rate of 50 mL/h with a syringe driver and a 1.30-mm diameter catheter tip during actual intra-arterial infusion, and a velocity of 0.01 m/s was prescribed at the catheter tip. The Reynolds number at the catheter tip is 13 in both models. The Reynolds number at the inlet of the CCA is 564 in model A and 664 in model B. Therefore, both flow of the anticancer agent and flow of blood were assumed as laminar flows. The mass fraction of the anticancer agent (*Y*_*0*_) was set as 1 at the catheter tip and 0 at the inlet of the CCA. In the wall boundary conditions, both the vascular and catheter walls were regarded as rigid and the no-slip condition was applied. In the outlet boundary conditions, the 0D resistance model described above was applied to each of the outlets, including the ICA and branches of the ECA. Because the STA is ligated with the catheter during the catheterization, neither the blood nor anticancer agent flows into the STA; therefore, the STA outlet was treated as a wall surface.

### Wall shear stress

Wall shear stress was (WSS) calculated using CFD, and WSSs of the vessels (LA and carotid bifurcation) and catheter in IAC models (A1-C, B1-C) and SSIAC models (A4, B4) were evaluated.

## Results

**Fig. 9:**
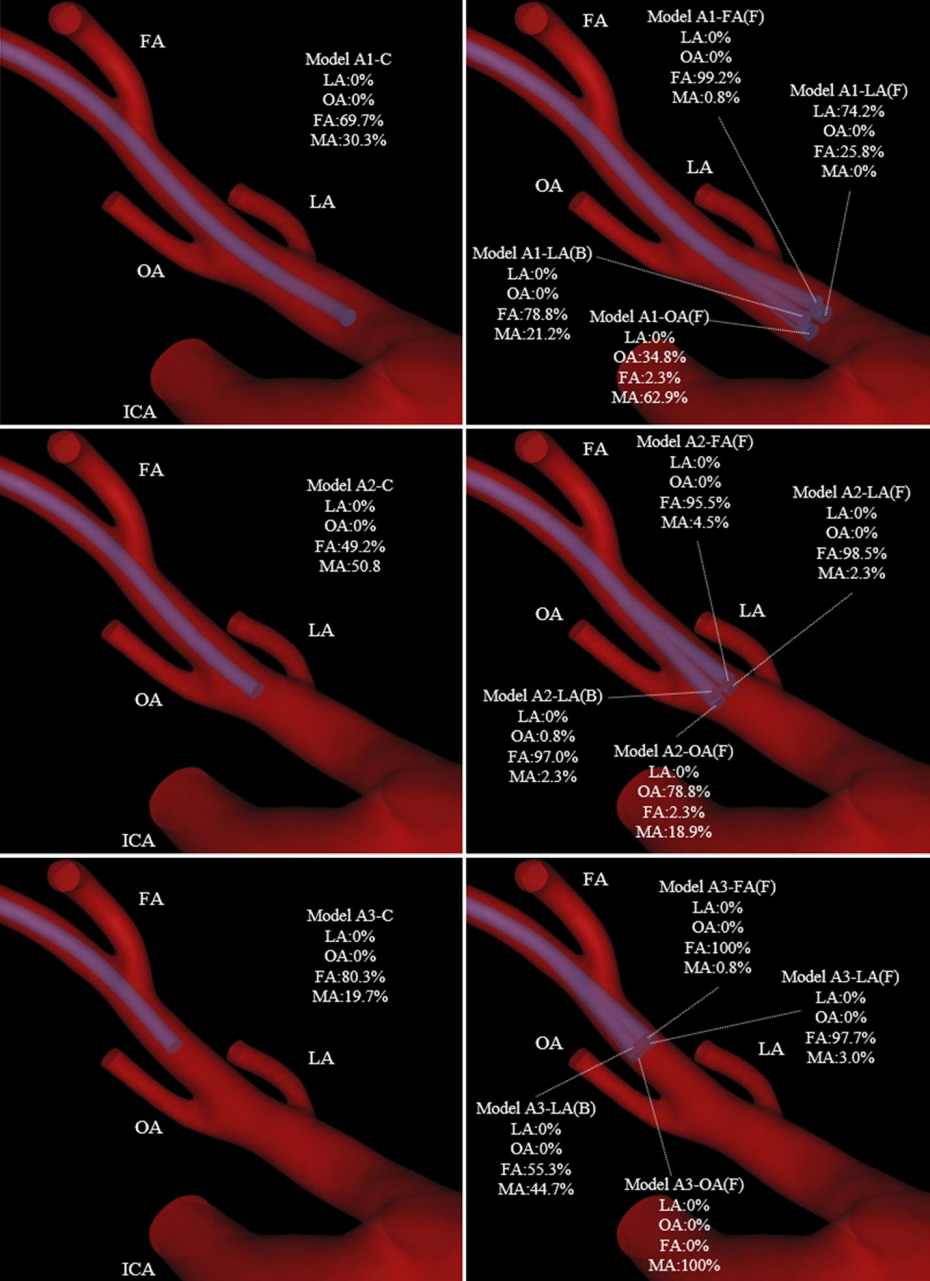
Distribution rates of the anticancer agent at each outlet in model A. *ECA* external carotid artery, *FA* facial artery, *ICA* internal carotid artery, *LA* lingual artery, *MA* maxillary artery, *MMA* middle meningeal artery, *OA* occipital artery

**Fig. 10:**
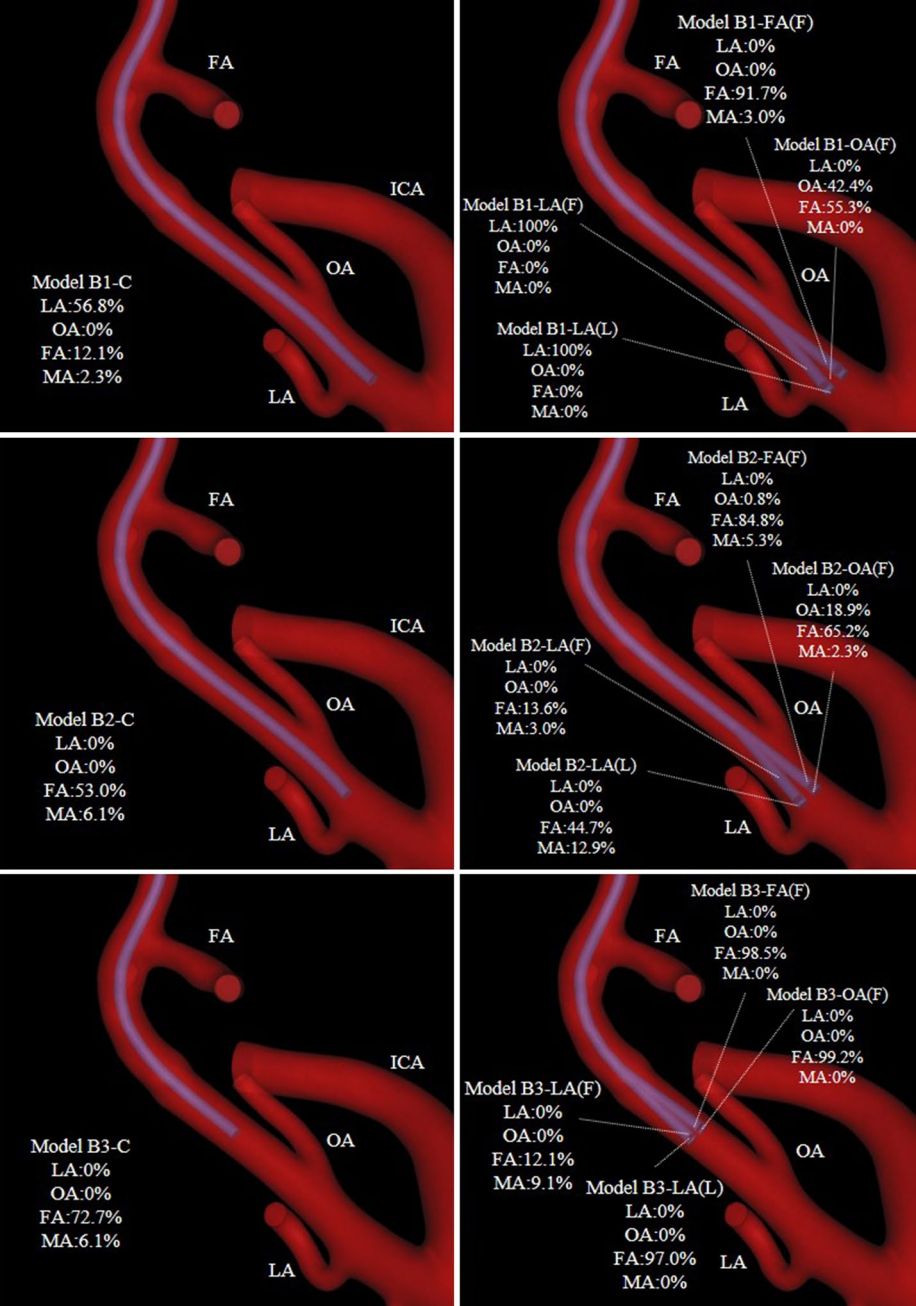
Distribution rates of the anticancer agent at each outlet in model B. *FA* facial artery, *ICA* internal carotid artery, *LA* lingual artery, *MA* maxillary artery, *OA* occipital artery

**Fig. 11:**
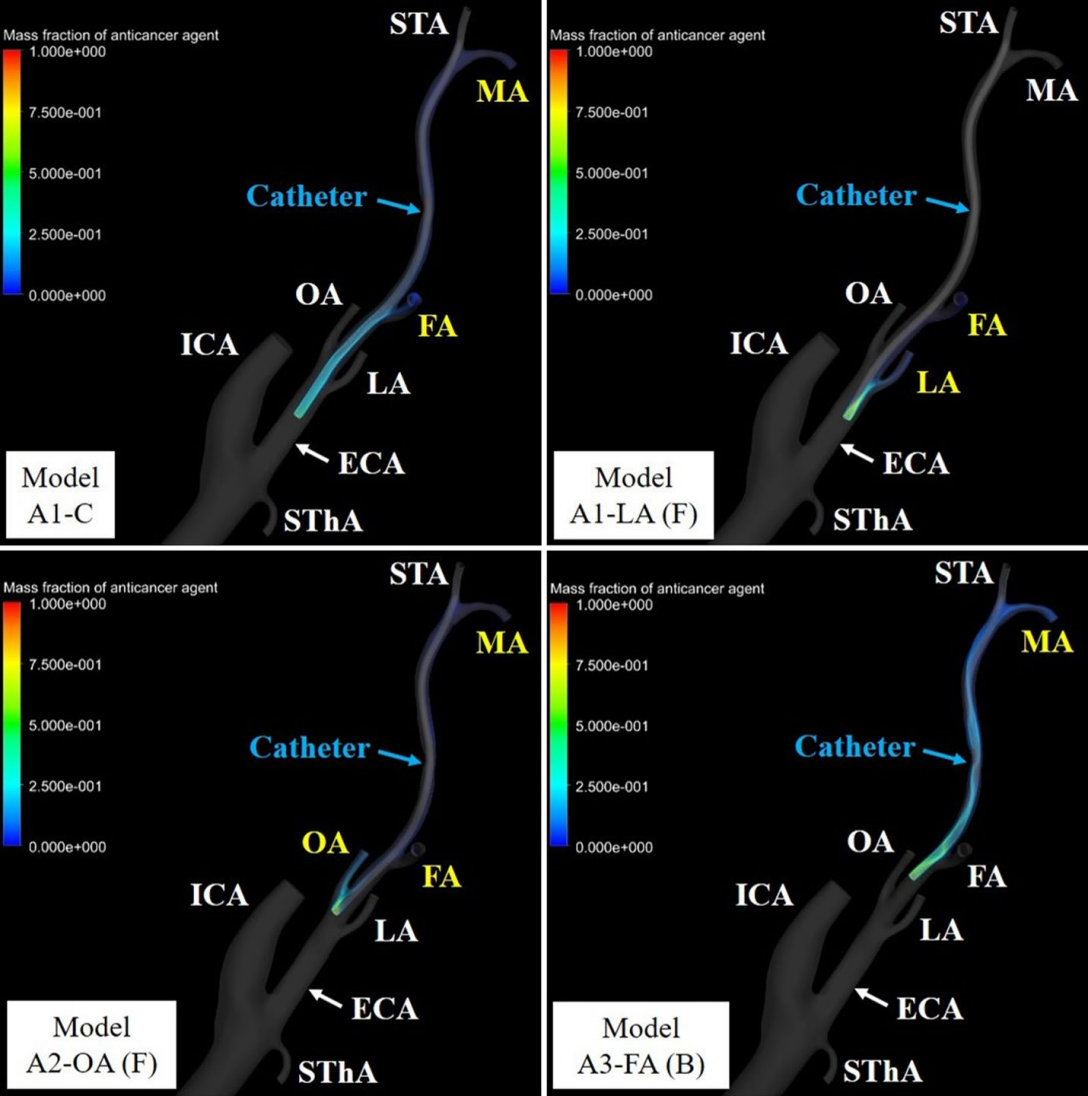
Volume rendering images of the mass fractions of the anticancer agent in model A. Branches of the external carotid artery (ECA) with flow of anticancer agent are shown in* yellow* type. *FA* facial artery, *ICA* internal carotid artery, *LA* lingual artery, *MA* maxillary artery, *OA* occipital artery, *SThA* superior thyroid artery

**Fig. 12:**
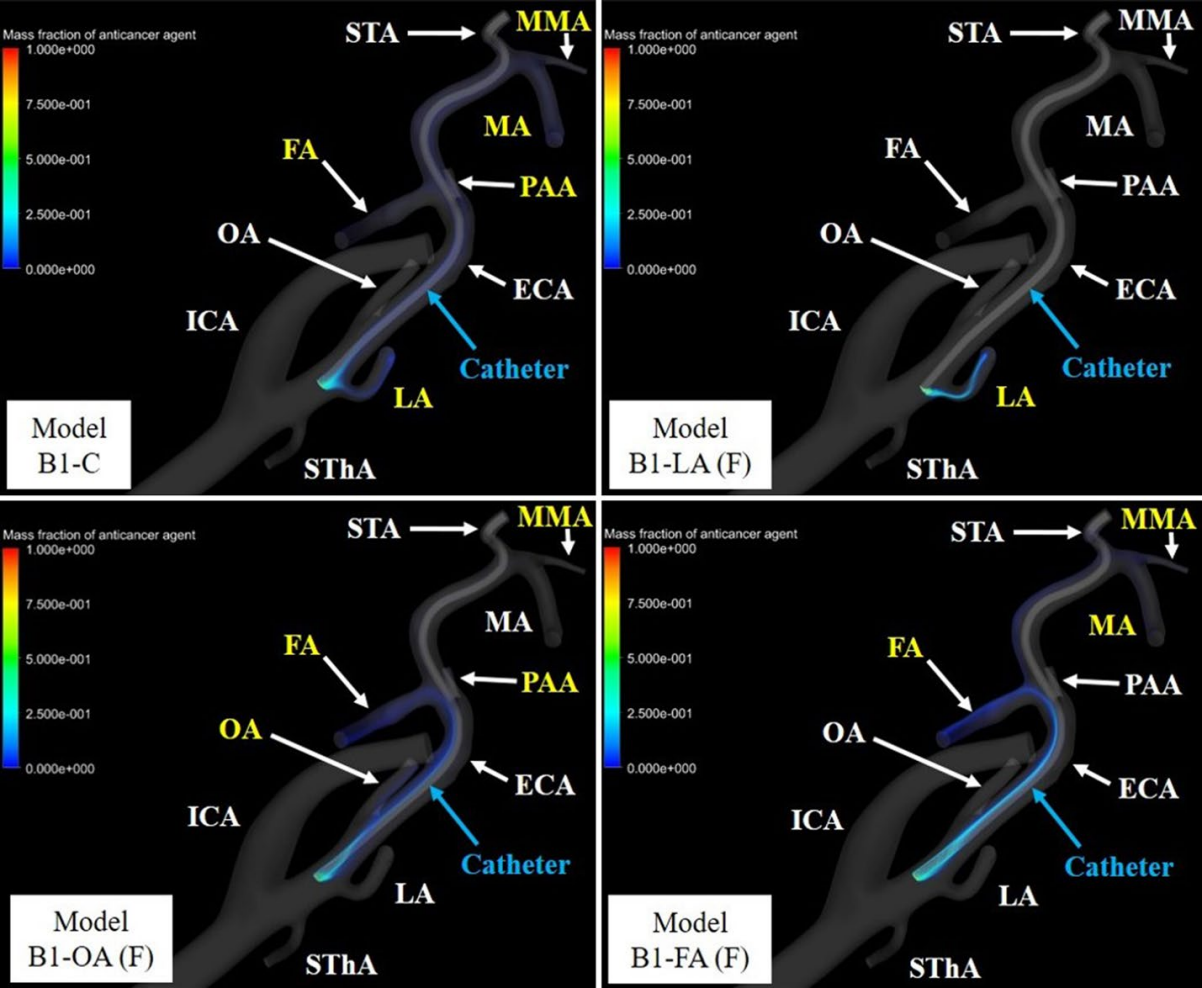
Volume rendering images of the mass fractions of the anticancer agent in model B. Branches of the external carotid artery (ECA) with flow of anticancer agent are shown in* yellow* type. *FA* facial artery, *ICA* internal carotid artery, *LA* lingual artery, *MA* maxillary artery, *MMA* middle meningeal artery, *OA* occipital artery, *PAA* posterior auricular artery, *SThA* superior thyroid artery

The simulations of vessel models A and B took approximately 40 h and 43 h to complete, respectively. The mass flow rates and distribution rates of the anticancer agent at each outlet (ECA branch) in 32 models are summarized in Tables 6 and 7. Figures 9 and 10 show the distribution rates of the anticancer agent on vessel images. Figures 11 and 12 show the flow of the anticancer agent in several models for the 2 cases. In several models for conventional IAC, the anticancer agent flowed into each target branch only when the catheter tip was located below the bifurcation between the ECA and each target branch. Furthermore, the anticancer agent tended to flow into the target branch when the catheter tip was shifted toward the target branch. In contrast, in models for SSIAC (models A4 and B4), the total amount of anticancer agent from the catheter tip flowed into the target artery.

**Table 6:** Mass flow rate (× 10^−6^ kg/s) and distribution rate of the anticancer agent in model A

Artery	Class 1					Class 2					Class 3					Class 4
	C	LA (F)	LA (L)	LA (R)	LA (B)	C	LA (F)	LA (L)	LA (R)	LA (B)	C	LA (F)	LA (L)	LA (R)	LA (B)	
		OA (R)	OA (F)	OA (B)	OA (L)		OA (R)	OA (F)	OA (B)	OA (L)		OA (R)	OA (F)	OA (B)	OA (L)	
		FA (L)	FA (B)	FA (F)	FA (R)		FA (L)	FA (B)	FA (F)	FA (R)		FA (L)	FA (B)	FA (F)	FA (R)	
ICA	0 (0%)	0 (0%)	0 (0%)	0 (0%)	0 (0%)	0 (0%)	0 (0%)	0 (0%)	0 (0%)	0 (0%)	0 (0%)	0 (0%)	0 (0%)	0 (0%)	0 (0%)	0 (0%)
SThA	0 (0%)	0 (0%)	0 (0%)	0 (0%)	0 (0%)	0 (0%)	0 (0%)	0 (0%)	0 (0%)	0 (0%)	0 (0%)	0 (0%)	0 (0%)	0 (0%)	0 (0%)	0 (0%)
LA	0 (0%)	9.8 (74.2%)	0 (0%)	0 (0%)	0 (0%)	0 (0%)	0 (0%)	0 (0%)	0 (0%)	0 (0%)	0 (0%)	0 (0%)	0 (0%)	0 (0%)	0 (0%)	13.2 (100%)
OA	0 (0%)	0 (0%)	4.6 (34.8%)	0 (0%)	0 (0%)	0 (0%)	0 (0%)	10.4 (78.8%)	0 (0%)	0.1 (0.8%)	0 (0%)	0 (0%)	0 (0%)	0 (0%)	0 (0%)	0 (0%)
FA	9.2 (69.7%)	3.4 (25.8%)	0.3 (2.3%)	13.1 (99.2%)	10.4 (78.8%)	6.5 (49.2%)	13.0 (98.5%)	0.3 (2.3%)	12.6 (95.5%)	12.8 (97.0%)	10.6 (80.3%)	12.9 (97.7%)	0 (0%)	13.2 (100%)	7.3 (55.3%)	0 (0%)
MA	4.0 (30.3%)	0 (0%)	8.3 (62.9%)	0.1 (0.8%)	2.8 (21.2%)	6.7 (50.8%)	0.3 (2.3%)	2.5 (18.9%)	0.6 (4.5%)	0.3 (2.3%)	2.6 (19.7%)	0.4 (3.0%)	13.2 (100%)	0.1 (0.8%)	5.9 (44.7%)	0 (0%)

The name of the ECA branch with a letter F, B, L, or R in bracket shows the horizontal position between the branch origin and catheter tip. Moreover, in both case A and B, the origin of LA, FA and OA located at angle with about 90 in distal view (Fig. 2). Each model has three names except models with horizontal position “C” or vertical position “Class 4″. For instance, LA (F), OA (R), and FA (L) mean the same horizontal catheter position in each class

*ICA* internal carotid artery, *FA* facial artery, *LA* lingual artery, *MA* maxillary artery, *OA* occipital artery, *SThA* superior thyroid artery

**Table 7:** Mass flow rate (×10^−6^ kg/s) and distribution rate of the anticancer agent in model B

Artery	Class 1					Class 2					Class 3					Class 4
	C	LA (F)	LA (L)	LA (R)	LA (B)	C	LA (F)	LA (L)	LA (R)	LA (B)	C	LA (F)	LA (L)	LA (R)	LA (B)	
		OA (L)	OA (B)	OA (F)	OA (R)		OA (L)	OA (B)	OA (F)	OA (R)		OA (L)	OA (B)	OA (F)	OA (R)	
		FA (B)	FA (R)	FA (L)	FA (F)		FA (B)	FA (R)	FA (L)	FA (F)		FA (B)	FA (R)	FA (L)	FA (F)	
ICA	0 (0%)	0 (0%)	0 (0%)	0 (0%)	0 (0%)	0 (0%)	0 (0%)	0 (0%)	0 (0%)	0 (0%)	0 (0%)	0 (0%)	0 (0%)	0 (0%)	0 (0%)	0 (0%)
SThA	0 (0%)	0 (0%)	0 (0%)	0 (0%)	0 (0%)	0 (0%)	0 (0%)	0 (0%)	0 (0%)	0 (0%)	0 (0%)	0 (0%)	0 (0%)	0 (0%)	0 (0%)	0 (0%)
LA	7.5 (56.8%)	13.2 (100%)	13.2 (100%)	0 (0%)	0 (0%)	0 (0%)	0 (0%)	0 (0%)	0 (0%)	0 (0%)	0 (0%)	0 (0%)	0 (0%)	0 (0%)	0 (0%)	13.2 (100%)
OA	0 (0%)	0 (0%)	0 (0%)	5.6 (42.4%)	0 (0%)	0 (0%)	0 (0%)	0 (0%)	2.5 (18.9%)	0.1 (0.8%)	0 (0%)	0 (0%)	0 (0%)	0 (0%)	0 (0%)	0 (0%)
FA	1.6 (12.1%)	0 (0%)	0 (0%)	7.3 (55.3%)	12.1 (91.7%)	7.0 (53.0%)	1.8 (13.6%)	5.9 (44.7%)	8.6 (65.2%)	11.2 (84.8%)	9.6 (72.7%)	1.6 (12. 1%)	12.8 (97.0%)	13.1 (99.2%)	13.0 (98.5%)	0 (0%)
PAA	0.3 (2.3%)	0 (0%)	0 (0%)	0.2 (1.5%)	0 (0%)	0.1 (0.8%)	0 (0%)	0 (0%)	0.1 (0.8%)	0 (0%)	0 (0%)	0 (0%)	0 (0%)	0 (0%)	0 (0%)	0 (0%)
MA	0.3 (2.3%)	0 (0%)	0 (0%)	0 (0%)	0.4 (3.0%)	0.8 (6.1%)	0.4 (3.0%)	1.7 (12.9%)	0.3 (2.3%)	0.7 (5.3%)	0.8 (6.1%)	1.2 (9.1%)	0 (0%)	0 (0%)	0 (0%)	0 (0%)
MMA	3.5 (26.5%)	0 (0%)	0 (0%)	0.2 (1.5%)	0.8 (6.1%)	5.3 (40.2%)	11.0 (83.3%)	5.7 (43.2%)	1.7 (12.9%)	1.4 (10.6%)	2.8 (21.2%)	10.5 (79.5%)	0.4 (3.0%)	0.1 (0.8%)	0.2 (1.5%)	0 (0%)

*ICA* internal carotid artery, *FA* facial artery, *LA* lingual artery, *MA* maxillary artery, *MMA* middle meningeal artery, *OA* occipital artery, *PAA* posterior auricular artery, *SThA* superior thyroid artery

**Fig. 13:**
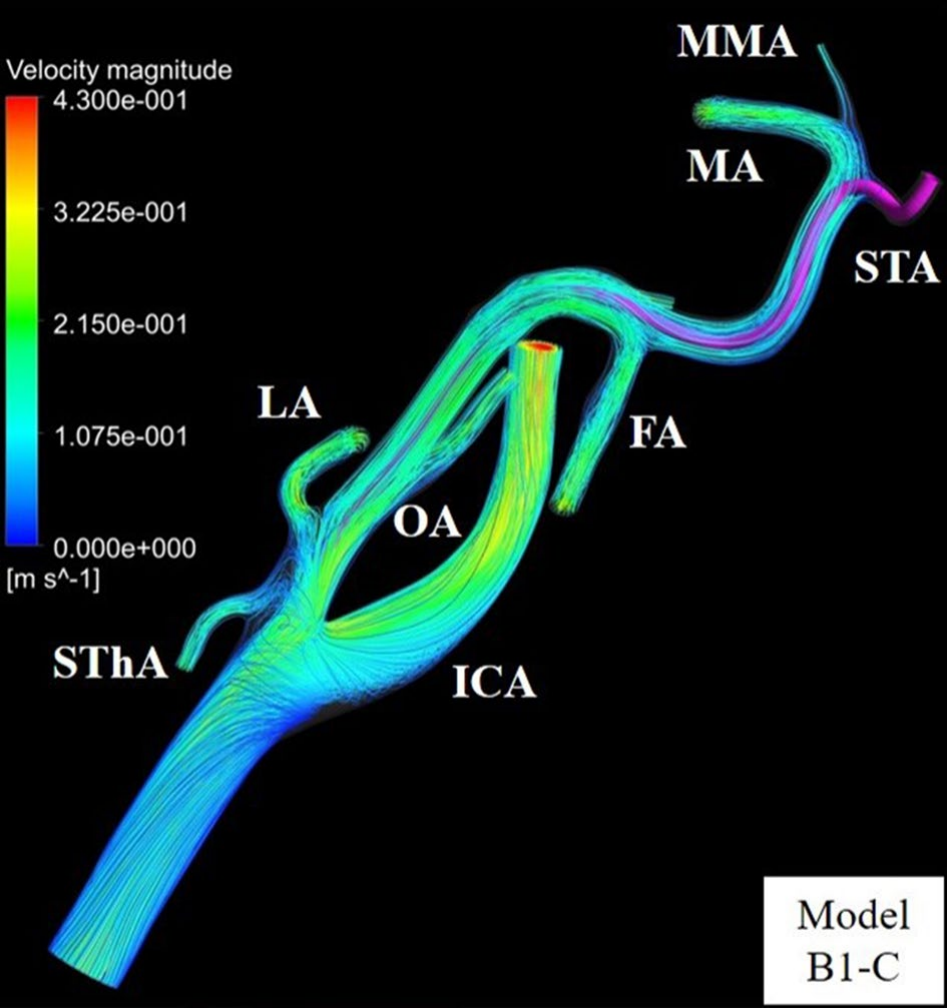
Blood streamline to target branches of the external carotid artery in model B1-C. Blood flow throughout the whole arterial system is shown by streamline. *ECA*, external carotid artery; *FA*, facial artery; *ICA* internal carotid artery, *LA* lingual artery, *MA* maxillary artery, *MMA* middle meningeal artery, *OA* occipital artery, *STA* superficial temporal artery, *SThA* superior thyroid artery

**Fig. 14:**
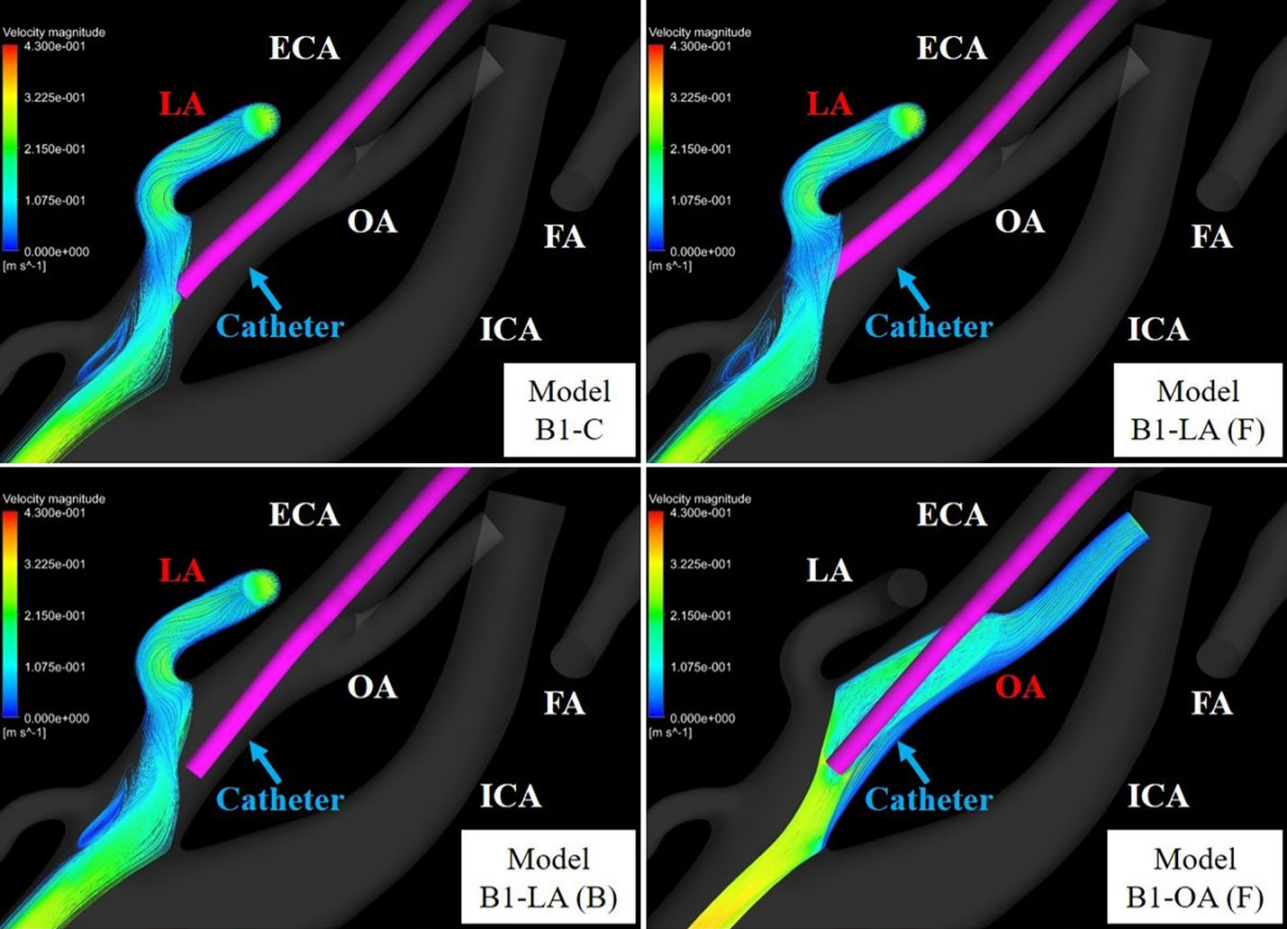
Blood streamline to target branches of the external carotid artery in model B. Blood flow to target branches (*red* type) is shown by streamline. *ECA* external carotid artery, *FA* facial artery, *ICA* internal carotid artery, *LA* lingual artery, *OA* occipital artery

To examine the flow of the anticancer agent in all models, the blood streamlines were traced from the inlet of the CCA toward each outlet. The streamlines of 4 models for model B are shown in Figs. 13 and 14. In all ECA branches that had flow of anticancer agent, the blood streamlines to the target branches contacted the catheter tip.

In models A1-LA (F), B1-C, B1-LA (F), and B1-LA (L) with flow of anticancer agent into the LA, blood streamlines toward the LA contact the catheter tip. In contrast, in model B1-LA (B), which did not show a flow of anticancer agent into the LA, the blood streamline toward the LA did not contact the catheter tip. Flow of anticancer agent to the OA was revealed in models A1-OA (F), A2-OA (F), B1-OA (F), and B2-OA (F). Anticancer agent flowed into the LA or OA only when the catheter tip was placed below the origin of each tumor-feeding artery, and it tended to flow into the target branch when the catheter tip was shifted toward the target branch. In contrast, flow of anticancer agent to the FA was shown in most models (27 of 30) for conventional IAC. In class C with the catheter placed at the center of the ECA, class 3 in which the vertical position of the catheter tip was closest to the origin of the FA provided high distribution rates (72.7–80.3%) of anticancer agent in both models A and B. Moreover, forward and backward shifts of the catheter tip in relation to the FA provided distribution rates of anticancer agent that were high (84.8–100%) and low (0–13.6%), respectively. However, unlike in simulations of anticancer agent flow into the LA and OA, the distribution rate of anticancer agent to the FA tended to increase as the vertical position of the catheter tip became closer to the origin of the FA when the tip was shifted right or left toward the FA; the mean distribution rates in classes 1-3 were 45.1% (range, 0–78.8%), 76.4% (range, 44.7–98.5%), and 87.3% (range, 55.3–99.2%), respectively.

**Fig. 15:**
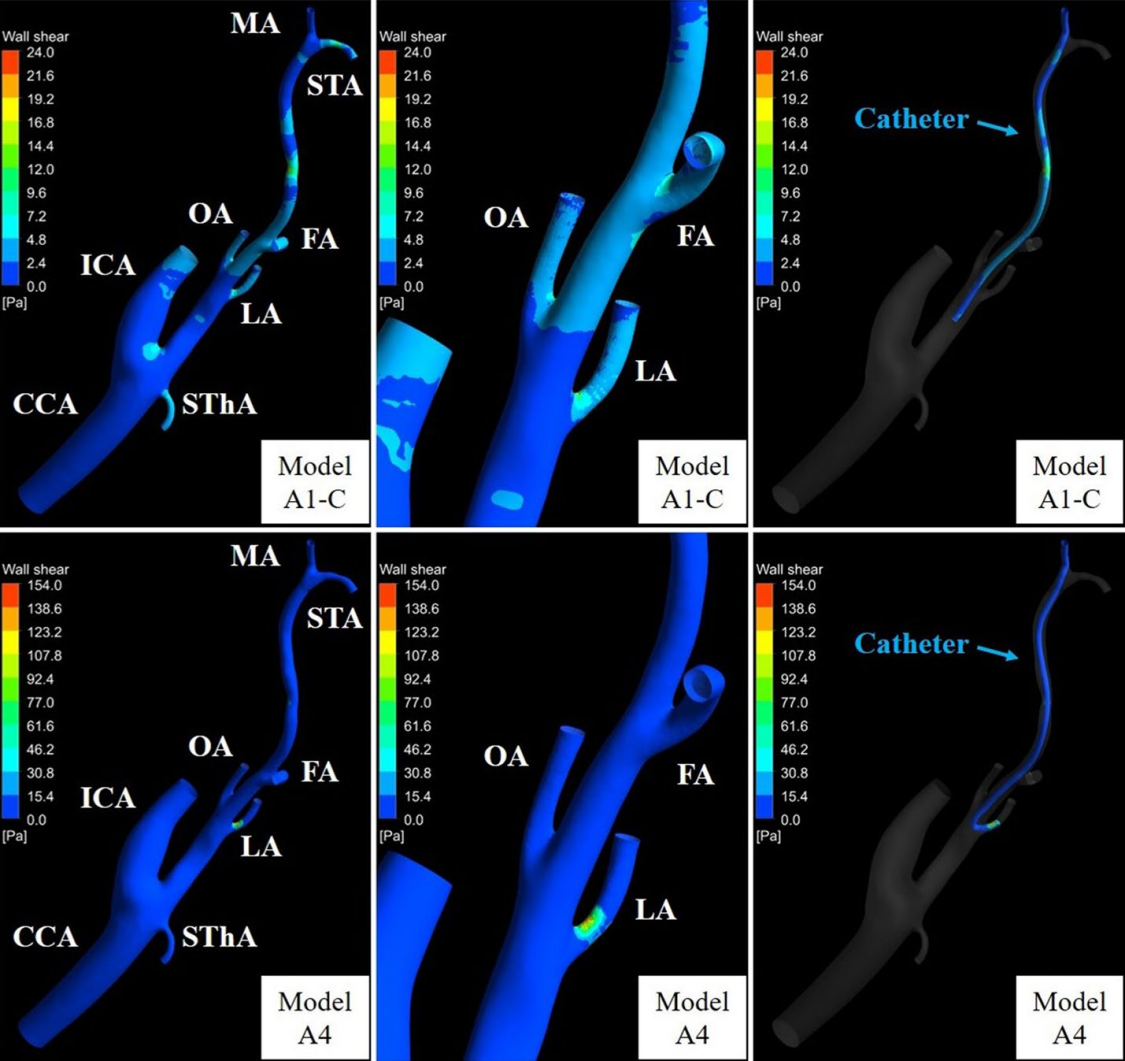
Wall shear stress distribution in models A1-C and A4. *FA* facial artery, *ICA* internal carotid artery, *LA* lingual artery, *MA* maxillary artery, *OA* occipital artery, *STA* superficial temporal artery, *SThA* superior thyroid artery

**Fig. 16:**
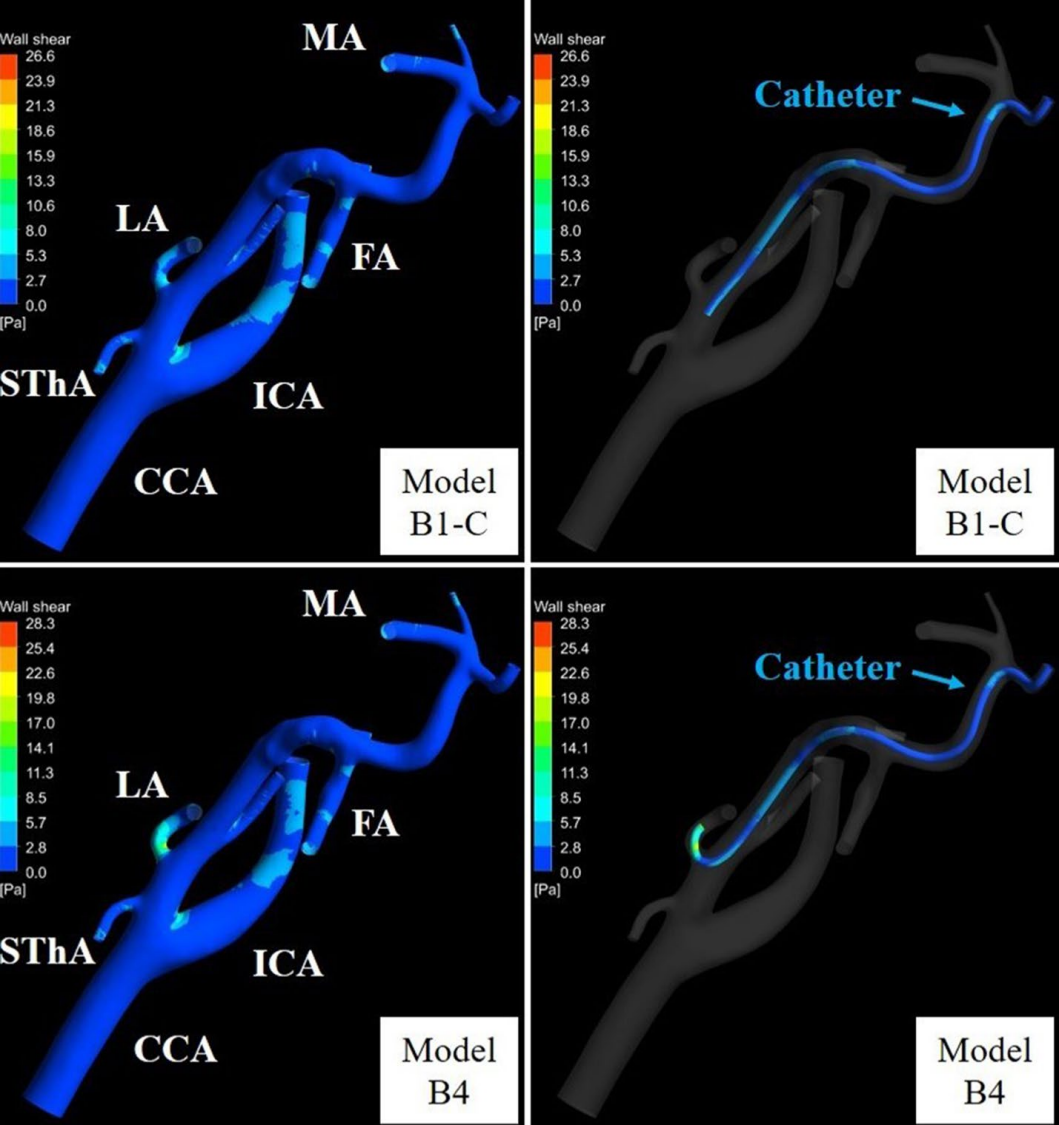
Wall shear stress distribution in models B1-C and B4. *FA* facial artery, *ICA* internal carotid artery, *LA* lingual artery, *MA* maxillary artery, *OA* occipital artery, *STA* superficial temporal artery, *SThA* superior thyroid artery

Figures 15 and 16 show WSSs of the vessel and catheter in models A1-C, A4, B1-C, and B4. In models A1-C and A4, the WSS values of the LA were 21.1 and 154.0 Pa, respectively. In models B1-C and B4, the WSS values of the LA were 12.7 and 22.7 Pa, respectively. The WSS values of the carotid bifurcation, which is the site of predilection for arteriosclerosis, in models A1-C, A4, B1-C, and B4 were 9.5, 9.9, 17.3, and 17.2 Pa, respectively.

## Discussion

Common feeding arteries of oral cancer include the LA, FA, and MA, which are branches of the ECA. Generally, a blue dye such as indigo carmine is manually injected to confirm whether the catheter is properly placed in the ECA in the vicinity of the tumor-feeding artery during catheterization or when IAC is performed. However, the high pressure created by manual injection can cause dye to reach the tumor even if the catheter is displaced from the origin of the tumor-feeding artery [3]. Administration of anticancer agents is performed not with a manual one-shot injection involving high pressure, but with a bolus or continuous infusion using a syringe driver; therefore, anticancer agents injected into the ECA may not reach the tumor-feeding artery [3]. The efficacy of conventional IAC for oral cancer is thus unproven and is not universally accepted [1, 3].

Because the tumor-feeding artery of maxillary sinus cancer or gingival cancer is the MA with/without the FA, the tip of the catheter is placed via the STA, mainly below the origin of the MA and occasionally below the origin of the FA. Catheterization below the origin of the MA in IAC allows anticancer agent flow to the MA that is similar to that in SSIAC because the catheter is ligated with the STA. Therefore, the present CFD study focused on the distribution of flow of anticancer agent into the branches of the ECA, excluding the MA, using several catheter positions for IAC.

To date, there have been few patient-specific blood flow simulations for IAC of head and neck cancer, including oral cancer. Rhode et al. [8] reported a simulation of hemodynamic flow in IAC using Seldinger’s method for head and neck cancer. Although a patient-specific vessel model was created from CT images, branches of the ECA, such as the OA, MA, and STA, were not created and a common trunk (linguofacial trunk), which is not a typical branch shape of the ECA [9, 19–21], was used. Furthermore, the catheter was placed at the CCA, which is not done in clinical practice, and the distribution rates of the blood flow, which were derived from other studies, were fixed at each outlet as the boundary condition, and the peripheral vascular network was not considered. Therefore, their CFD analysis was not conducted in a patient-specific manner and no physiological pressure was provided.

The present CFD study applied patient-specific vessel models from CT images and a 0D resistance model, with estimation of vessel pressure and flow distribution as a boundary condition, for patients with tongue cancer fed by the LA. Only model LA (F) in class 1 of model A allowed flow of the anticancer agent into the LA. However, flow of the anticancer agent was observed in three models in class 1 of model B. In particular, all anticancer agents injected from the catheter flowed into the LA in models B1-LA (F) and B1-LA (L). Flow of anticancer agent to the OA was revealed in models A1-OA (F), A2-OA (F), B1-OA (F), and B2-OA (F). Anticancer agent flowed into the LA or OA only when the catheter tip was placed below the origin of each tumor-feeding artery, and it tended to flow into the target branch when the catheter tip was shifted toward the target branch. As shown in Fig. 14, the present CFD study revealed that the anticancer agent can flow into the tumor-feeding artery in conventional IAC when the blood streamline toward the tumor-feeding artery from the CCA contacts the catheter tip. These IAC simulation results suggest that the catheter tip should be placed both below and toward the target artery to increase the distribution rate of anticancer agent into the LA and OA. In contrast, flow of anticancer agent to the FA was shown in most models for conventional IAC. However, contrary to flow simulations of anticancer agent into the LA and OA, the distribution rate of anticancer agent to the FA tended to increase as the vertical position of the catheter tip became closer to the origin of the FA when the tip was shifted right or left toward the FA. Because the cross-sectional area of the ECA gradually decreases toward peripheral arteries, such as the STA, and the blood streamline toward the FA from the CCA tends to contact the catheter tip, the distribution rate of anticancer agent to the FA tends to increase as the catheter tip becomes closer to the origin of the FA.

Branches of the ECA have anatomical variations, including the common trunk or the order of branch origins. The respective incidence of thyrolingual trunk and linguofacial trunk has been reported as 0.7–3% [19–21] and 2.7–31% [19–22]. In a cadaver study, Yonenaga et al. [21] reported the incidence of order of the ECA branches (observed from the caudal side) as follows: (1) LA, FA, and OA in 41.1% of cases; (2) LA, OA, and FA in 17.6%; (3) OA, LA, and FA in 7.1%; (4) linguofacial trunk and OA in 21.4%; (5) OA and linguofacial trunk in 10.7%; and (6) thyrolingual trunk, FA, and OA in 1.9%. In the present study, the order of ECA branches in both model A and model B (from the caudal side) was LA, OA, and FA. The present flow simulation suggests that flow distribution of anticancer agent into the FA might decrease when the FA originates from the ECA between the LA and OA, because the cross-sectional area of the ECA increases as the origin of the FA becomes closer to the carotid bifurcation and the blood streamline toward the FA from the CCA tends not to contact the catheter tip. However, the distribution rate of anticancer agent into the FA may be increased by placement of a curved catheter tip both below and toward the target artery.

Wall shear stress is associated with endothelial function and is calculated by CFD. For an oral cancer patient, Rhode et al. [8] simulated the WSS of the CCA bifurcation in the case of Seldinger’s method using CFD. The WSS of the carotid bifurcation found this way was high (80 Pa) because the catheter was placed at the CCA below the carotid bifurcation and injection velocity was high (2.5 mL/s). Because high WSS can potentially cause acute vascular endothelial changes, the present study evaluated WSSs in IAC and SSIAC models. The WSS of the LA in model A4 (154.0 Pa) was much higher than that in model A1-C (21.1 Pa) because reduction of blood flow into the small-diameter LA by insertion of the catheter caused a large velocity gradient of the flow near the catheter. As the acute yield stress of the vessel is reported to be approximately 37.9 Pa [23], SSIAC may cause acute vascular endothelial changes at the LA in case A. In contrast, the WSS values of the LA were less than 37.9 Pa in both models B1-C and B4 (12.7 and 22.7 Pa, respectively). This is because the diameter of the LA (about 3.0 mm) was larger than in case A. Therefore, conventional IAC, as well as SSIAC, may not cause acute vascular endothelial changes at the LA in case B. This WSS simulation can allow the method of catheter placement to be selected preoperatively to avoid damaging the tumor-feeding artery with the catheter.

There were some limitations in the present study. This study assumed a healthy blood vessel, but angiogenesis has been observed in regions of tumor progression. Therefore, it may be not reasonable to assume that the parameters of vessels in the tumor region are similar to those of healthy blood vessels and further studies should be performed in the future. Pulsatile flow simulation with unsteady analysis is also needed to simulate distribution of the anticancer agent and WSS of the ECA for the whole duration of infusion (1 h). Furthermore, the vascular wall was considered to be a no-slip rigid wall in this study. Because vascular elasticity can affect the flow of blood and anticancer agent in actual IAC, fluid structure interaction analysis is necessary to account for the elasticity of the vessel walls and catheter movement. Only two patient-specific datasets were used in this study, so additional simulation with more patients’ data will be needed to understand the characteristics of anticancer agent flow in the ECA branches of patients.

## Conclusions

We numerically investigated the flow distribution of anticancer agent into the branches of the ECA in IAC models. The CFD simulation results of IAC showed that a curved catheter tip may be placed both below and toward the target artery to increase the distribution rate of anticancer agent into the tumor-feeding artery. Although SSIAC can reliably supply anticancer agent to the target artery, high WSS at the target artery can occur, depending on vessel geometry of the patient, and this may cause serious complications during treatment. In future study, analysis of the fluid structure interaction is necessary to account for the elasticity of the vessel walls and catheter movement in many patient models.

## Abbreviations

0D: zero-dimensional
3D: three-dimensional
CCA: common carotid artery
CFD: computational fluid dynamics
CT: computed tomography
DICOM: Digital Imaging and Communications in Medicine
ECA: external carotid artery
FA: facial artery
IAC: intra-arterial chemotherapy
ICA: internal carotid artery
LA: lingual artery
MA: maxillary artery
MMA: middle meningeal artery
OA: occipital artery
PAA: posterior auricular artery
SIMPLE: semi-implicit method for pressure-linked equations
SSIAC: superselective intra-arterial chemotherapy
STA: superficial temporal artery
SThA: superior thyroid artery
STL: Standard Triangulated Language
